# Combining evidence and values in priority setting: testing the balance sheet method in a low-income country

**DOI:** 10.1186/1472-6963-7-152

**Published:** 2007-09-24

**Authors:** Emmanuel Makundi, Lydia Kapiriri, Ole Frithjof Norheim

**Affiliations:** 1 National Institute for Medical Research, Dar es Salaam, Tanzania; 2 University of Toronto Joint Centre for Bioethics, Toronto, Ontario, Canada; 3 Centre for International Health, University of Bergen, Norway; 4 Division for Medical Ethics, Department of Public Health and Primary Health Care, University of Bergen, Norway

## Abstract

**Background:**

Procedures for priority setting need to incorporate both scientific evidence and public values. The aim of this study was to test out a model for priority setting which incorporates both scientific evidence and public values, and to explore use of evidence by a selection of stakeholders and to study reasons for the relative ranking of health care interventions in a setting of extreme resource scarcity.

**Methods:**

Systematic search for and assessment of relevant evidence for priority setting in a low-income country. Development of a balance sheet according to Eddy's explicit method. Eight group interviews (n-85), using a modified nominal group technique for eliciting individual and group rankings of a given set of health interventions.

**Results:**

The study procedure made it possible to compare the groups' ranking before and after all the evidence was provided to participants. A rank deviation is significant if the rank order of the same intervention differed by two or more points on the ordinal scale. A comparison between the initial rank and the final rank (before deliberation) showed a rank deviation of 67%. The difference between the initial rank and the final rank after discussion and voting gave a rank deviation of 78%.

**Conclusion:**

Evidence-based and deliberative decision-making does change priorities significantly in an experimental setting. Our use of the balance sheet method was meant as a demonstration project, but could if properly developed be feasible for health planners, experts and health workers, although more work is needed before it can be used for laypersons.

## Background

Tanzania, as many other countries in sub-Saharan Africa, needs to improve the way resources are allocated in health care. Around US$8 are spent per capita on health care per year, so hard policy decisions have to be made which affect millions of people [1]. Several building blocks are now in place to help improve the decision-making process. First, the Ministry of Health has adopted the strategy of identifying a set of core health care services in what they call an 'essential health care intervention package' [2]. Although this package is defined in broad and somewhat unspecific terms, it provides a good starting point for allocating resources efficiently and fairly. Second, the country has embarked upon an ambitious health sector reform, involving decentralised planning and financing through basket funds at regional and district levels. By bringing decision making closer to the people affected, decentralised planning is thought to facilitate sensitivity to local priorities and public involvement [3]. Third, the Ministry has set up a National Sentinel Surveillance System in collaboration with the Adult Mortality and Morbidity project. The aim of this was to provide timely and accurate information on local and national burden of disease profiles so as to improve evidence-based decision making process [4]. Finally, despite scarcity of evidence on clinical outcomes and the cost-effectiveness of actual interventions, there is an increasing body of high quality studies on clinical outcomes and cost-effectiveness relevant for Tanzania or East-Africa. Through the WHO CHOICE project, more evidence on the cost-effectiveness of interventions will become available [5].

At the international level, researchers, political decision-makers and organisations acknowledge the need to develop methods for using evidence in resource allocation which are efficient and seen as fair by the public [6-9]. The goal of allocative efficiency is based on the notion that health resources should be allocated across interventions and population groups to generate the highest possible overall level of population health [10]. Cost-effectiveness information is critical in defining a mix of interventions that would maximise health in the absence of any constraints on possible decisions except a finite budget. However, there are other influences on decisions, such as political constraints (including dominant interest groups), donor agencies' priorities, multiple levels of government and dysfunctional financing systems (not further discussed in this paper). In addition there are distributional concerns, and concerns about public acceptability [7]. The last two are requirements of fairness. Fairness requires that health benefits should be distributed according to some notion of equity, and that the public perceives this distribution as fair [11].

From a health policy perspective, much is known about the requirements of allocative efficiency. However, there is less agreement on how to develop political procedures which secure the requirements of fairness [12]. The issue of legitimacy has been addressed from two broad perspectives. The first derives its ideas from participatory democracy which requires the involvement of the public in priority setting [13-17]. The second derives its ideas from deliberative democracy and ideas of public accountability. This argues that procedures can be considered fair if they satisfy certain conditions, such as publicity, transparency, the provision of relevant reasons for not providing services, the existence of proper appeal mechanisms, and the regulation of the process [11,18,19]. However, research and development of procedures for resource allocation have not identified agreed methods for identifying, assessing and validating the evidence that decisions are based on. There is, therefore, a need for a combined approach, which acknowledges the crucial importance of assessing evidence on clinical outcomes and evidence on cost-effectiveness, and which, at the same time, incorporates the requirements of legitimacy and fairness. In particular, key research issues to consider when testing a priority setting method that combines scientific evidence and societal values may include: identification of the priorities of a selected sample of stakeholders, whether provision of evidence affects the respondents' priorities, whether deliberation and voting processes change participants' priorities, if respondents' rankings are in any way different from ranking according to cost-effectiveness, and finally whether the method is feasible for incorporating both scientific evidence and societal values into priority setting processes in various contexts including resource-poor settings.

This study was motivated by these concerns. We aimed at testing out the balance sheet method as a model for incorporating scientific evidence and societal values in priority ranking of nine health care interventions in a setting of extreme resource scarcity.

## Methods

**Figure 1:**
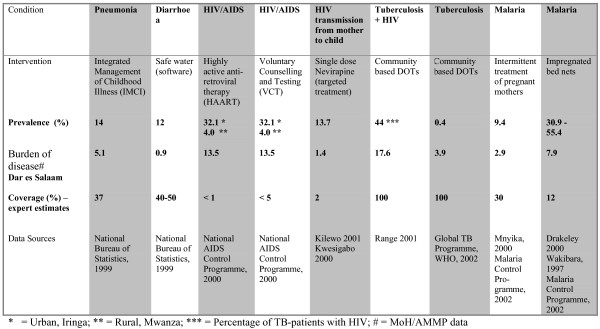
Evidence presented to the participants. Balance sheet 1. Estimates of need: Prevalence, burden of disease and coverage.

**Figure 2:**
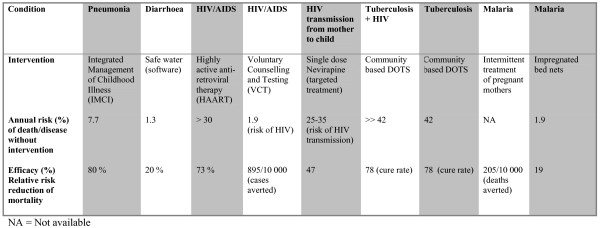
Evidence presented to the participants. Balance sheet 2. Clinical outcomes.

**Figure 3:**
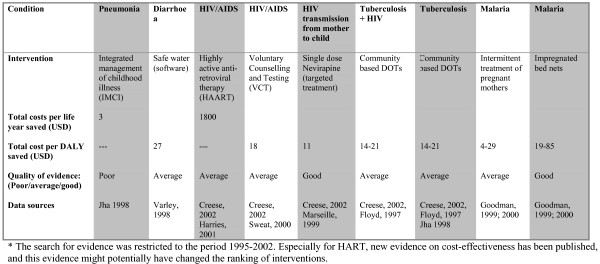
Evidence presented to the participants. Balance sheet 3. Cost-effectiveness. The search for evidence was restricted to the period 1995–2002. Especially for HART, new evidence on cost-effectiveness has been published, and this evidence might potentially have changed the ranking of interventions.

**Figure 4:**
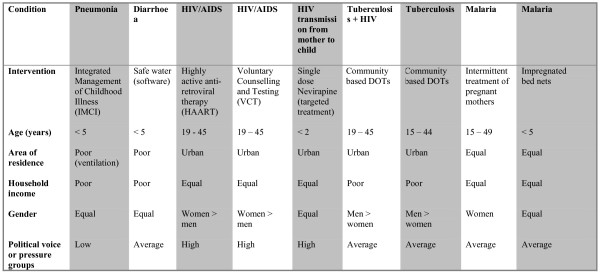
Evidence presented to the participants. Balance sheet 4. Equity considerations.

In collaboration with local experts in epidemiology and health planning, we identified six conditions and nine interventions relevant for an essential health care intervention package in Tanzania. These interventions included Integrated Management of Childhood Illness (IMCI), Safe Water (software, i.e. educational tools, not plumbing etc.), Highly Active Anti-Retroviral Therapy (AIDS), Voluntary Counselling and Testing (HIV), single dose Nevirapine (prevention of HIV from mother to child), Community Based DOTS (for patients with tuberculosis and HIV), Community Based DOTS (tuberculosis only), Intermittent Treatment of Pregnant Mothers (malaria), and Impregnated Bed Nets (malaria). Before conducting group interviews with key stakeholders, we collected several types of evidence in four steps (see figures 1, 2, 3, 4).

The types of evidence included were all based on criteria that have been proposed as relevant in the literature on priority setting, such as prevalence, disease burden, coverage, severity of disease (annual risk of death or disease without intervention), efficacy (relative risk-reduction of mortality), cost-effectiveness, and equity [16]. These criteria are not derived from a unified framework of criteria for priority setting, but were selected because we wanted to test whether they were also considered relevant for our respondents.

### Step I: Assessment of evidence on prevalence, disease burden and coverage of the selected conditions

The Global Burden of Disease Study and the East African Regional Study provided data on incidence and prevalence [20]. A systematic search on Medline was added to develop best estimates on incidence and prevalence [21-29]. We also contacted national experts for the selected conditions, for validation of our data and asked them to give their own estimates based on their experience in the various fields. They also gave us local estimates for the coverage of the selected interventions. The local organisations contacted included relevant departments at the Ministry of Health (the National Malaria Control Programme, the National TB Control Programme, the Integrated Management of Child Illness Programme, the National AIDS Control Programme), and the Adult Mortality and Morbidity Project (AMMP), as well as key organisations such as the Tanzanian Essential Health Care Intervention Project (TEHIP), and the regional WHO office. From AMMP we received updated burden of disease estimates for Dar es Salaam region and Hai district, the closest approximation of disease burden in the nearby Moshi district [see Additional file 1] [30,31].

### Step II and III: Review of evidence on cost-effectiveness of selected interventions

We conducted a systematic search in Medline and the Cochrane database for studies on cost-effectiveness for all the identified health interventions. We used the search terms: 1) "condition/intervention", 2) "cost*effectiveness", and 3) "Tanzania" or "sub-Saharan Africa", for the period of 1995–2002. The search term for country was broadened if no records were found. Preference was given to studies where details of costs and benefits were clearly presented and where the outcome measures were either life years or disability adjusted life years saved – studies used: [32-43]. We did not conduct a separate search on clinical outcomes, but identified randomised clinical trials and meta-analyses used in the actual cost-effectiveness studies.

### Step IV: Critical review of literature on equity and political concerns in the distribution of health benefits

Finally, we undertook a critical review of the literature on health policy and equity issues in the distribution of health benefits, from which we developed a list of group characteristics of the target population relevant to equitable health policy [16,44,45]. The list included age, area of residence, household income, gender, and political voice. We described each target group according to this list, and the validity of these descriptions was itself a topic for discussion in the group interviews (see study procedure, step 6).

The information obtained was presented in an explicit and neutral manner to the respondents, in the form of a Balance Sheet, developed according to David Eddy's method [46]. The criteria used in the balance sheet method overlaps considerably with those used by Eddy, but the researchers made the final decision about the relevant criteria.

#### Sampling

Through the National Institute for Medical Research, we identified eight groups of stakeholders in priority setting in health, of which four were from an urban setting (Dar es Salaam), and four from a rural setting (Moshi). These included representatives from the general population, health workers, national and district planners (of the Health Sector) and one patient group (people living with HIV/AIDS). We obtained informed consent from all participants. Each group comprised of eight to thirteen adult participants:

##### 1. Moshi District

i) Moshi District Health Management Team (DHMT) members (8 participants)

ii) Mawenzi hospital health workers (doctors and nurses) (13 participants)

iii) People living with HIV/AIDS (10 participants)

iv) Lay people from Makuyuni village (10 participants)

##### 2. Dar es Salaam

i) Temeke Municipal Health Management Team members (13 participants)

ii) Lay people from Chamazi village (11 participants)

iii) Muhimbili National Hospital health workers (doctors and nurses) (10 participants)

iv) Ministry of Health officials (10 participants).

#### The modified nominal group interview

We conducted an interview study using the nominal group technique [47]. The original nominal group interview can be described as a focus group interview with voting on key issues [48]. We employed a modified nominal group technique. Our modification consisted of introducing evidence in a stepwise manner and allowing more discussion of conflicting issues before voting. The use of votes is thought to overcome the usual unequal representation of opinions. This method is considered to be better than focus groups for generating ideas and getting equal participation from group members.

### Study procedure

Each group interview lasted about 3–4 hours. Each group session began with self-introductions, introduction of the purpose of the interview, as well as discussion of the selected conditions and their interventions to ensure a common understanding. After this, with the facilitation of the investigators, each group went through eight sessions:

1. Participants were asked to rank the interventions without any evidence and prior to any discussions. The question asked: "Please rank the interventions according to your personal view of perceived importance. As more resources become available, they will be used according to the rank order given."

2. After this, participants were requested to imagine they were health planners interested in the population health of their society. The question asked: "Imagine now that you are health planners: How would you rank the interventions?" The group discussed the interventions presented and came up with a priority ranking of the interventions by the whole group, through consensus, or if necessary through voting.

3. Presentation of estimates of prevalence, burden of disease, and coverage (balance sheet 1) followed by individual ranking.

4. Presentation of information on clinical effectiveness (balance sheet 2) followed by individual ranking.

5. Presentation of information on cost- effectiveness of the interventions (balance sheet 3) followed by individual ranking.

6. Presentation of group characteristics for each condition (balance sheet 4). The respondents were asked to discuss the validity of this information and its relevance for equity considerations. They were then asked to do the final individual ranking based on all the evidence given.

7. Participants were asked to imagine they were health planners again. They discussed the interventions and came up with a priority ranking of the interventions by the whole group, through consensus or voting.

8. Brief evaluation of the methodology and summing up.

All group discussions were actively facilitated and participants had opportunities to ask for clarifications. Presentation of the evidence (the wording and use of technical terms such as risk reduction or odds ratios) varied according to the informant's level of expertise. Where appropriate, discussions were carried out in Kiswahili. All participants gave their written informed consent, and ethical and scientific clearance for this study was obtained from Medical Research Co-ordinating Committee of the National Institute for Medical Research of Tanzania.

## Analysis

We collected each respondent's ranking before any evidence was given, and after introduction of the four kinds of evidence outlined above. All the individual rankings were tabulated into a spreadsheet, and we calculated mean rankings from each group and for all participants. Eight groups were convened with a total of 85 respondents, but three responses (two from the village group), had to be excluded because of obvious misunderstandings and inconsistencies. We present descriptive statistics for the remaining 82 respondents. The two rankings obtained through consensus or voting before and after all the evidence were tabulated separately, and the mean for all groups was calculated (representing average rankings for all groups after deliberation). While aggregation of the ranks masks the individual- and group-differences, the purpose of this paper was to test the application of the tool. Detailed discussion of the differences between individual groups will be presented elsewhere.

For comparison of different rankings, we defined a rank deviation as significant if the rank order of the same intervention differed by two or more points on the ordinal scale. Difference in rank order is expressed as a ratio between the interventions with significant rank deviation over the total number of interventions.

## Results

The main result from the sample of stakeholders in Tanzania, as expressed after all the evidence was received and discussed, is that Integrated Management of Childhood Illness (IMCI) should be considered the first priority, followed by Voluntary Counselling and Testing (VCT) and Prevention of Vertical Transmission of HIV from mother to child with single dose Nevirapine (both ranked as number two). The three interventions with the lowest priority were intermittent treatment of pregnant mothers with malaria (rank seven), followed by Directly Observed Treatment for tuberculosis (DOTS) for patients with the combined diagnosis of tuberculosis and HIV. Highly Active Anti-Retroviral Therapy (HAART) for HIV/AIDS was ranked lowest (table 1).

**Table 1:** Final ranking after deliberation and voting, mean rank, all groups

Final ranking after deliberation and voting	
IMCI	1
Voluntary Counselling and Testing (VCT)	2
Nevirapine (prevention of HIV from mother to child)	2
DOTs (TB alone)	4
Bed nets (malaria)	5
Safe water	6
Intermittent treatment with SP (malaria)	7
DOTs (+ HIV)	8
Anti-retroviral therapy (HAART)	9

**Figure 5:**
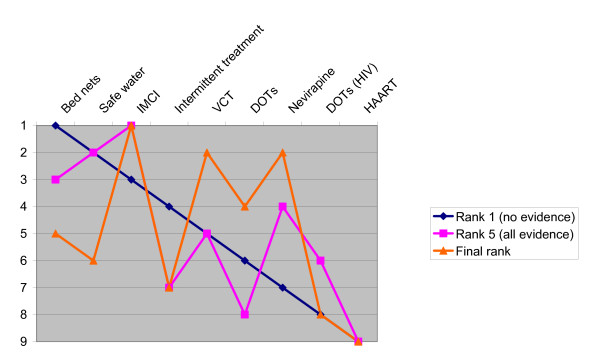
Relative ranking of interventions. Comparison of rank order before and after evidence was provided, and after the final deliberation and voting.

Our study procedure made it possible to compare the groups' ranking before and after all the evidence was given (figure 5). Based on comparisons of rank, the difference between the initial rank versus the final rank (before discussion and voting) was a rank deviation of 67% (6/9). We also tested whether deliberation and voting changed the rank order. The difference between the initial rank versus the final rank after discussion and voting was a rank deviation of 78% (7/9).

**Figure 6:**
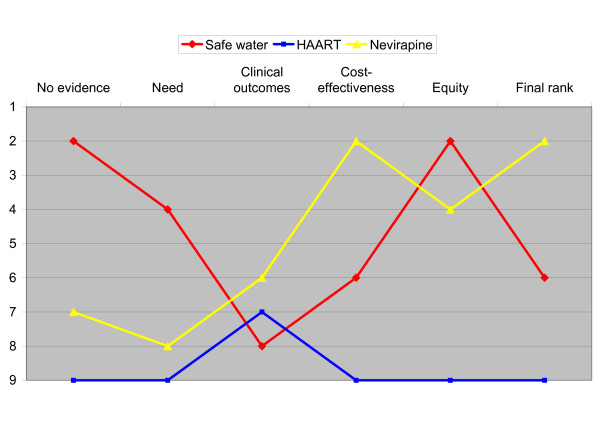
Relative ranking of interventions. How evidence and deliberation change the relative rank of three selected interventions.

From our data, we were also able to explore how evidence and deliberation changed the rank order of various interventions. Three examples are given in figure 6. Software for safe water, e.g. educational tools, was initially considered second priority. When evidence of need was presented (life years lost from diarrhoea is reported to be about 1% of the total burden of disease), the rank dropped, and dropped further when the mortality reduction of 20% was presented. Ranking improved somewhat when the cost-effectiveness of $27 per life year saved was considered, and the intervention resumed its rank number two when the respondents considered that diarrhoea is a condition typically affecting poor people with a low political voice. In the overall discussion, the relative rank dropped to number six. Highly Active Anti-Retroviral Therapy (HAART) was consistently given lowest priority, except when the favourable clinical outcomes were examined. The prevention of vertical transmission of HIV from mother to child with Nevirapine got a low rank initially, but improved significantly to rank number two when the favourable cost-effectiveness ratio (11$ per DALY saved) was presented.

**Figure 7:**
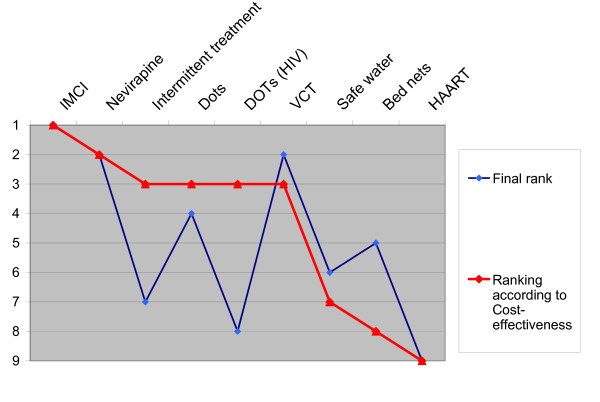
Relative ranking of interventions. Final rank compared with ranking according to cost-effectiveness ratios.

Although ranking interventions according to data from different studies in a league table form is methodologically problematic, we wanted to test how the participants' rank order compared with such a league table (figure 7). The league table is based on median point estimates of cost-effectiveness, even if some of the studies used expressed interval estimates. We found that the respondents' rankings differed only slightly from ranking according to cost-effectiveness. Rank deviation was 33% (3/9).

The most marked deviations were for Intermittent Treatment with sulfadoxine-pyrimethamine (SP) for pregnant women to prevent malaria, DOTS for patients with tuberculosis and HIV, and Insecticide-treated Bed Nets. In discussions, respondents gave various reasons for their ranking. For Intermittent Treatment with SP, many experts expressed concerns about the lack of evidence from randomised clinical trials on the assumed mortality reduction from this intervention (a fact acknowledged in the original cost-effectiveness study). DOTS for patients with TB and HIV was given low priority since, as some said, life expectancy for these patients is shorter because of lack of access to anti-retroviral therapy in the country. Others supported the current TB policy in Tanzania (which assigns equal priority to all with TB regardless of HIV status). The Impregnated Bed-Nets strategy was given high priority by many because malaria is the major cause of under-five mortality, and participants felt that an intervention addressing this problem was important.

**Figure 8:**
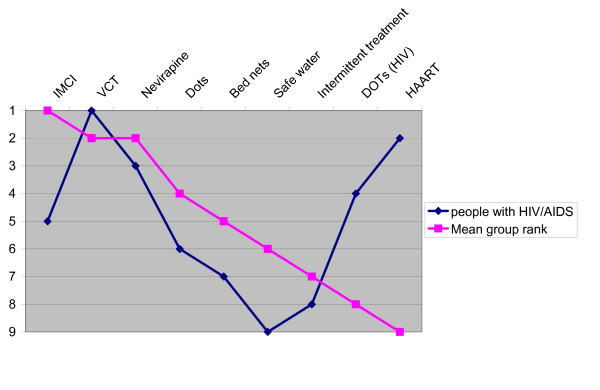
Relative ranking of interventions. Ranking from the patient group (people living with HIV/AIDS).

Is the balance sheet method feasible for involving patients and the public? Our results from the patient group are somewhat discouraging in this respect (figure 8). Patients living with HIV/AIDS consistently ranked all interventions related to HIV on top, despite all the evidence given. It is, however, interesting to observe that voluntary counselling and testing (VCT) got a clear priority over HAART, even if this preferences would not favour patients' own condition as much as HAART. This might indicate that overwhelming evidence on the cost-effectiveness of VCT contributes to reasonableness in open discussion.

The interviews in the village groups (n = 21) gave valuable inputs, but in analysing the material we found that many participants found the ranking process difficult and we had to exclude two of the individual rankings because they were inconsistent. In the evaluation, many participants said that they found the information too technical to comprehend. Two of the participants could not read, and many had difficulties with numbers. We found the balance sheet approach more relevant and useful for health workers and planners.

## Discussion

Our study suggests that evidence-based and deliberative decision-making does change priorities significantly in an experimental setting in a low-income country. This confirms findings from UK [49]. Informed decision-making with the use of the balance sheet approach is feasible for health planners, experts and health workers, but can be less so for laypersons. The study demonstrates some of the strengths of the balance sheet method. It brings evidence and other reasons to the table and provides a common arena for debate. It helps sort out relevant arguments, and encourages critical assessment of evidence so that reasons for rationing can be made explicit.

There is partial overlap with the criteria our respondents found important and those found in a very interesting discrete choice experiment from Ghana [50]. That study was carried out to determine the relative importance of different criteria in identifying priority interventions in Ghana and found that interventions that are cost-effective, reduce poverty, target severe diseases, or target the young had a higher probability of being chosen than others. Our study was not designed to identify the relative importance of each criterion and the results can therefore not be directly compared.

There are several weaknesses to the study. We presented realistic choices in a hypothetical choice situation. The exercise is hypothetical in two ways: First, we did not present all available evidence to the participants. The amount of potentially relevant information is much larger than the selection we chose. In this sense, we limited the reasons participants could take into consideration. Second, policy makers must typically compare more than a few interventions at a time (the WHO-CHOICE database project aims to produce data for about 500 interventions). One of the major shortcomings of the balance sheet method is that its priority ranking results seem to be limited to the interventions included in the study design. Results will obviously change when other alternatives are introduced. However, all methods for priority setting that rely on evidence and comparisons of alternatives are incomplete if they do not include all relevant alternatives. Starting with a limited set of interventions and making the evidence explicit is only the first step for comprehensive priority setting. At a later stage more interventions and types of evidence can be included. What our study shows is that discussion of evidence and values may change priorities, and this way of making the process more transparent may be an advantage also in real-world situations where the alternatives are many and complex.

Moreover, the choice situation we created was highly context sensitive. For example, we reported that coverage for treatment of smear positive patients with tuberculosis is almost 100 percent. This might have influenced the priority assigned to treatment for this condition (i.e. coverage is already almost complete).

We did not ask respondents about re-allocations, and that this could be seen as a major drawback of the tool. However, there is nothing in the tool itself that prohibit rephrasing the question posed to the respondents – so that re-allocation is also considered.

The search for evidence was restricted to the period 1995–2002. Especially for HAART, new evidence on cost-effectiveness has been published since, and this evidence might have changed the ranking of interventions. A less narrow search strategy could also have changed our results, i.e. we could have included studies from outside sub-Saharan Africa, and cost-effectiveness studies with other outcome measures than life years or DALYs. Moreover, we presented evidence on clinical outcomes based on results from meta-analyses and studies used in key cost-effectiveness studies. Performing a systematic search and full evaluation of all possibly relevant clinical studies for all nine interventions is extremely resource and time demanding, and we therefore assumed that key studies had already been identified in this way in the meta-analyses and the economic studies we used. However, our simplified approach could have introduced biases that the reader should be aware of.

With regards to the composition of the respondent groups, we chose homogenous groups because given the cultural-, social- and gender- norms in this society; dominance of educated males may have been unavoidable. Homogeneous groups are typically considered better to avoid dominance from single individuals with expert status.

Although the respondents gave reasons for their ranking and these would add understanding of the rankings, we do not report analysis of them here. This paper is the first exploration of this tool and its application. Details of the reasons and discussions during the deliberation will be presented elsewhere.

In the evaluation, the respondents expressed that they did not have enough time to understand all the information. The balance sheet is difficult for laypersons and non-medical planners to understand, but even experts in the fields complained about the complexities of choice: four sets of evidence for nine interventions had to be balanced at the same time. On the other hand, it is hard to see how to reduce the complexities of this kind of decision-making. Many expressed the need for capacity building with respect to summarising the evidence base. Finally, some expressed concern that the discussion and voting procedure was easy to manipulate, eloquent rhetoricians tended to influence the consensus, or the open voting. We could have used secret ballots.

Overall, the study suggests that many of the arguably most important decisions in priority setting – such as the assessment and interpretation of evidence – are so technical that they are not feasible for direct participation from the public. However, the balance sheet promotes what we will call internal accountability through explicitness, transparency and a commitment to scientific validity. This fits more easily into the deliberative framework advocated by among others Daniels and Sabin, focusing on accountability for reasonableness [11]. Requirements other than participation are probably needed for securing external accountability, that is, accountability to the public.

## Conclusion

The results from our exploratory study concerning ranking should not be generalized as an expression of all stakeholders' views for policy decisions in Tanzania. We do, however, think that the balance sheet method indicates some elements which are needed to improve resource allocation processes in Tanzania. As a first step, there is a need for institutional strengthening in terms of capacity building for the identification, assessment and interpretation of available evidence. The methods and tools of evidence-based medicine provide a useful starting point for such capacity building [51]. In addition, there is a relative scarcity of evidence from randomized clinical trials and cost-effectiveness studies relevant for this area. Strengthening national research involvement and building capacity to make use of the WHO-CHOICE databases is also important. With these building blocks in place, health authorities at all levels would have the opportunity to improve health policy through informed debate. Although there's increasing recognition of the relevance of involving the public in priority setting, the limitations of their ability to decipher medical evidence has made it difficult to implement. This study provides a method that could facilitate meaningful public involvement in priority setting. However, there is need for more research to explore how this would play out in real life planning situations.

## Competing interests

The author(s) declare that they have no competing interests.

## Authors' contributions

LK and OFN conceived of the study, and EM, LK and OFN participated in its design and coordination and helped to draft the manuscript. All three authors participated in the group interviews and data analysis. All authors read and approved the final manuscript.

## Pre-publication history

The pre-publication history for this paper can be accessed here:



## Supplementary Material

Additional file 1 Burden of Disease in Hai and Moshi distrcts. Click here for file
