# Supplementary material for: Combining evidence and values in priority setting: testing the balance sheet method in a low-income country
Source: BMC Health Serv Res. 2007 Sep 24;7:152. doi: 10.1186/1472-6963-7-152 (PMC2096625; doi:10.1186/1472-6963-7-152)
Supplement: Additional file 1 — Burden of Disease in Hai and Moshi distrcts. [file 1472-6963-7-152-S1.doc]

# Appendix B: Burden of Disease data presented to the participants
